# The Horizon of Unintentional Injuries among Children in Low-Income Setting: An Overview from Bangladesh Health and Injury Survey

**DOI:** 10.1155/2009/435403

**Published:** 2009-08-23

**Authors:** S. M. Chowdhury, A. Rahman, S. R. Mashreky, S. M. Giashuddin, L. Svanström, L. G. Hörte, F. Rahman

**Affiliations:** ^1^Division of Social Medicine, Department of Public Health Science, Karolinska Institutet, 171 76 Stockholm, Sweden; ^2^Centre for Injury Prevention and Research, Bangladesh (CIPRB), Mohakhali, Dhaka 1207, Bangladesh

## Abstract

*Introduction*. The paper aims to explore the magnitude and distribution of unintentional injuries among Bangladeshi children (<18 years). *Methodology*. A cross sectional survey was conducted during 2003 (January to December) in 12 randomly selected districts and Dhaka Metropolitan City of Bangladesh. Nationally representative data were collected from 171 366 households comprising of 351 651 children of under 18 years. Information includes the number of deaths and illness at the household in the preceding year. Verbal autopsy and verbal diagnosis form was used to determine the cause of mortality and morbidity respectively. *Results*. There were 351651 children in the study, of which 5577 had one or more injuries in the past one year. Drowning and falls was the leading cause of injury mortality and morbidity in children over 1 year of age respectively. Incidence of unintentional injuries was significantly higher among boys (95% CI = −2157.8) than girls (95% CI = 968.7 − 1085.8) while rural children were the most vulnerable group. Home and its premises was the most common place for the injury incidence. *Conclusion*. The result of the study could be an insight to the policy makers to develop realistic and effective strategies to address the issue.

## 1. Introduction

Worldwide, there is a consensus that low-and middle-income countries are passing through an epidemiological transition. The pattern of childhood mortality and morbidity is changing from infectious diseases to noncommunicable diseases and injuries in these countries. Although, childhood injuries are a major health problem worldwide, [1, 2] but our knowledge is inadequate about their epidemiology [3]. In children beyond the first few months of life, trauma is the most significant cause of mortality and disability, being responsible for more deaths than all diseases combined [4, 5]. In 1990, injuries in the developing countries contributed to 13% of total disability adjusted life years among children. It is expected that by 2020, this share will increase to 22% [6]. Fatal injuries are, however, only the tip of the iceberg, because for every child who dies many more will suffer nonfatal injuries with varying degree of disability. In 1999, injuries claimed the lives of 17 940 Americans younger than 20 years of age, which is more than all natural causes of death combined [7]. Unintentional injuries accounted for approximately 76% of all injuries in Hong Kong during 1998 in children age 1–14 [8].

Like other countries of epidemiological transition, gradual shift in the cause of child deaths from infectious disease to non-communicable disease and injury has been observed in Bangladesh [9]. Recent evidence from the Demographic Surveillance System of the International Centre for Diarrhoeal Disease Research, Bangladesh (ICDDR, B), shows a growing proportion of child deaths due to injuries [10]. In Bangladesh, there are only few studies [11, 12] that show the burden of injuries. However, most of the child health programs in Bangladesh are focused on prevention of infectious and nutritional causes of child death.

Without knowledge of the basic epidemiology of injuries, effective injury prevention and acute care cannot be carried out [13]. Thus, to develop a realistic unintentional child injury prevention program, in this study we aimed to explore the magnitude and distribution of unintentional injuries among Bangladeshi children.

## 2. Materials and Methods

A cross sectional survey was conducted during 2003 (January to December) in 12 randomly selected districts (out of 64 districts) and Dhaka Metropolitan City. The Survey used stratified, multistage, and cluster sampling design. The strata were administrative regions and rural and urban areas. With in strata, three-stage cluster sampling was performed. Simple random sampling was used to distribute sampling unit.

In Bangladesh, there are six divisions, so from each division two districts were selected by random sampling method. In each district, to represent the rural community, one upazlia (rural subdistrict, which is almost homogeneous in all districts) was randomly chosen and in each upazila two unions (administrative lowest units composed of approximately 20 000 people) were also selected randomly, and each union was considered as a cluster. All households in the union were included in the survey. The district headquarters of the 12 selected districts and Dhaka Metropolitan City constituted the urban areas. In the urban areas, mohallas (urban subunit—mohalla is a term to describe a neighborhood or locality in the cities and towns) served as clusters. By multistage cluster sampling data was collected from 171 366 households; 88 380 from rural areas, 45 183 from district towns (urban areas), and 37 803 households from Dhaka Metropolitan City. In the sampled households 351 651 children of 0–17 years were identified, 177 985 males and 173 666 females. This enormous sample size was required in order to provide sufficient power to capture the rare fatal child injury events.

Data were collected by 48 trained data collectors through face-to-face interviews with respondents. Along with the researchers, six fulltime supervisors were employed for supervision and monitoring the data collection process. Mothers were primarily preferred as respondents. However, if mother was not available the most knowledgeable members of the household were considered as respondents. Where possible, it was the head of household, and as many members of the household were present as possible to corroborate or add detail to the respondents' interview answers. Screening forms were used to identify any mortality or morbidity in the household. A household member was defined as member living in the same house including domestic helpers and long-term guests, sharing meals and information. The respondents were first asked if there had been any deaths or illness of children 0–17 years in the households in the preceding year. Repeated attempts were administered in case of unavailability of respondents on first visit. The detailed methodology of the survey has been published elsewhere [14].

After pretesting, three structured questionnaires were developed for data collection. Part I was screening form for identification of illnesses and deaths in the household including age and sex characteristics. Part II form was for collecting information on the socioeconomic characteristic. Part III form covered the detail information on death, due to injury, treatment history before death and postmortem history. Verbal autopsy method which is a valid instrument was used [15, 16] to determine the cause of mortality as well as verbal diagnosis form that was used for morbidity.

Operational definitions of different levels of injury severity used in this study are as follows.

**Moderate**

Sought medical care but not admitted to hospital; or had a three-day work loss or absence from school, but had no permanent disability. Three days were set as the minimum number following extensive discussions with social scientists and epidemiologists familiar with Bangladeshi cultural norms.

**Major**

Hospitalized for a period of less than 10 days but no permanent disability.

**Serious**

Hospitalized for 10 days or more, but no permanent disability.

**Severe**

Permanently disabled (loss of vision, hearing, handling, ambulation, or mentation) regardless of whether hospitalization occurred.

Data collected in this survey was entered into a data entry program developed by the investigators using Epi-Info 6. Data was double entered by two different groups and then merged for validation of data entry. After cleaning, the data was exported to SPSS 12.1 for analysis. Standard descriptive statistics were used to analyze the characteristics of unintentional injuries. The incidence was calculated with exact binomial 95% CI. Relative Risk (RR) was calculated to compare the risk of unintentional injuries in different age groups, place of residence, and sex.

## 3. Results

During the study period we identified 5577 cases of unintentional injuries in children aged 17 years or less including 154 fatal cases. Common causes of injuries among these children were falls (*n* = 1663; 29.8%), burns (*n* = 1013; 18.2%); injury by sharp cutting object or cut injury (*n* = 743; 13.3%); road traffic injury (RTI) (*n* = 675; 12.1%); drowning/near drowning (*n* = 495; 8.9%); animal bite (*n* = 361; 6.5%), and electrocution (*n* = 277; 5.0%). The rate of fatal and nonfatal injury among children of under 18 years was 43.8 per 100 000 children years and 1542.2 per 100 000 children years, respectively. Children below 1 year old were less vulnerable to injuries where as 1–4 years old children were the most vulnerable group. Drowning was the leading killer of children over 1 year of age and falls were identified as the leading cause of injury morbidity.

However, in the study falls, burn, cut injury and road traffic injury were found as the 4th, 5th, 7th, and 8th leading cause of burden of diseases in regard to morbidity among children over one year of old (Table 1).

The incidence of unintentional injuries was significantly higher among boys (2074.8 per 100 000 children-year; 95% CI = 1994.9–2157.8) than girls (1025.6 per 100 000 children-year; 95% CI = 968.7–1085.8). Besides, it was observed that incidence rate of injuries among rural children (2177.1 per 100 000 children-year; 95% CI = 2099.0–2285.1) were significantly higher than the urban children (843.9 per 100 000 children-year; 95% CI = 789.7–909.1). Table 2 illustrates the injury incidence rate by gender and geographic distribution.

**Figure 1:**
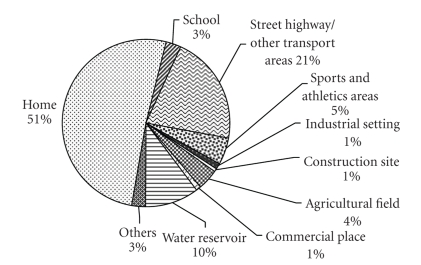
Place of unintentional injury occurrence in children 0–17 years of age.

Home and its premises were the most common place for injury incidence. However, highways/street, water reservoir, sports areas, agriculture field, and schools were the other common sites where various proportions of unintentional injuries among children. Injury incidence in different age groups by place of occurrence is showed in Figure 1.

Most of the children who sustained injury were involved in indoor playing (23.4%) at the time of incidence followed by working in the agriculture field (20.9%), outdoor playing (20.2%), and traveling (13.8%). Involvement of children in different activities prior to injury has been shown in Table 3.

**Figure 2:**
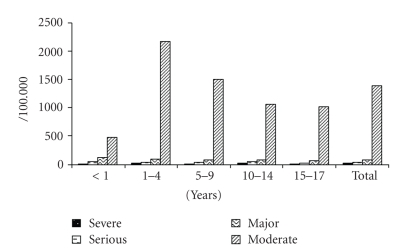
Distribution of unintentional injury severity in children 0–17 years by age groups.

During the study period 74 cases of severe injury, that is, permanent disability were identified with an incidence rate of 21 per 100 000 child-years. Injury was also found to be responsible for different durations of disability (Figure 2) as per injury severity. Falls were the leading cause of permanent disability due to injury among children followed by burn and cut injury.

### 3.1. Drowning

Drowning peaked in the 1–4 age group and then rapidly declined as age increased. The age pattern of near drowning was identical to that of drowning, only the rate was several times higher (118/100,000 near-drowning; 30/100,000 drowning) among children 1–17 years. Ponds were the most common place of childhood drowning in Bangladesh, and most of the drowned children could not swim.

### 3.2. Falls

Fall was the leading cause of injury morbidity and disability. Nonetheless, fall was the 4^th^ leading cause of morbidity after infancy and was responsible for 8.6% of all causes of morbidity. Around 57 percent of all falls occurred at home and its premises.

### 3.3. Burns

Burn due to flame (40%) was the main cause; cooking fire was the major source (58 percent) of flame. The flame of kerosene lamp was the other source of burn especially for infants (23 percent) and 1–4 years children (11 percent). Female children were mainly the victims of burn in older age groups by cooking fire. The hot liquid food, hot rice water, hot water for cooking, hot cooking oil, and hot water for bathing were the major causes of scalding. More than 33 percent of burn due to hot object was caused by charcoal and 16.4 percent by cooking utensils.

### 3.4. Road Traffic Injury

Road traffic injury was identified as one of the major causes of nonfatal injuries among children and was the second leading cause of nonfatal injury among children aged 15–17 years. Child pedestrians were the main victims of road traffic injuries (*n* = 267, 39.5%). Other less common types of victims were rickshaw passengers or passenger of the nonmotorized vehicle (*n* = 260; 38.5%); bus/minibus passengers (*n* = 55; 8.1%); motorcycle passengers (*n* = 22; 3.3%); truck passengers (*n* = 6; 0.9%).

### 3.5. Other Main Causes of Unintentional Injuries

Fatal animal bite rates were highest in mid childhood and late adolescence. The peak rate was 9.8/100,000 in 15–17 year old children. The fatal bites were all due to dogs and snakes, with an equal proportion of each, and all occurred in rural areas.

Cut injuries were almost uniform in all age groups after infancy, and the incidence rate was 204.7/100,000 among all children. However, cut injury was significantly higher in rural areas. About 40% of all cut injuries were caused by knives or knife-like cutting instruments.

In the study, no fatal poisoning case was identified during the survey period. The highest rate of nonfatal poisoning was in infants (24.7/1001,000) followed by children aged 1–4 years (21.6/1001,000). One-third of nonfatal accidental poisonings were caused by insecticides followed by pesticides and detergent.

During the survey, only five deaths were found due to electrocution among children, and all of them were males in rural areas. All these deaths were caused by exposed electric wires inside the house (80%) as well as outside the house (20%). The nonfatal electrocution rate among children under 18 years was 77.3/100 000. Electrocution had a rural predominance in all age groups with four or five times higher than urban rates.

## 4. Discussion

In this study, 351 651 children younger than 18 years of age were evaluated. The most common causes of injury in this study were falls, burns, cut injuries and transport injuries (RTI), and similar findings have been observed in several studies [17, 18] published elsewhere. While sex-specific injury incidence rates were calculated, maleś injury incidence rate was significantly higher than that of females. Surprisingly, almost every kind of injury in each age group male was at higher risk than female. This finding is not unusual [19]. The factors that lead to this increased risk for males are complex and difficult to untangle. They may include inborn differences in behavior, as well as obvious differences in exposure related to traditional male and female roles in our society. As a generalization, females spend much more time at home and do not go outside the home with the same frequency as males. Subtle differences in socialization operating at a very early age may produce gender-dependent differences in risk taking [20]. In our study we found that burn was the leading cause of injury among younger children below 5 years though drowning was the leading killer in this age group. A hospital-based study in Ghana also revealed that burn was the leading cause of injury in that age group [21]. Besides, our study also established fall as the leading cause of injury among the children of 5–17 years. Similar findings were also observed in different studies conducted elsewhere in the world [22] though some other studies showed that transport injuries were the most frequent cause of injury [21, 23].

In this study, major differences in frequency of injuries between urban and rural residents observed, perhaps because of the hazards of agricultural machinery, chemicals, and exposed bodies of water. Similar findings have been observed in other population-based survey, [24]. This has great implications in setting priorities when planning for intervention strategies.

Home was found as the commonest place where more than 50% of all injuries occurred, followed by street/highway and water reservoir. The importance of the home environment in the causation of injuries has been recognized by many authors in this field [25, 26]. The more hazards there are in the home, the more likely it is for the child to get injuries. Thus, parents and caregivers should make their homes safe for young children as it is impossible to supervise children 24 hours.

This study established the significant higher incidence of drowning in rural settings; possibly this difference is most likely due to the differences in urban and rural environments regarding water sources. Most of the drowned children could not swim. Since, an effective intervention [27] swimming learning program could be introduced to prevent drowning, though children of 1–4 years were the main victims.

The pattern of poisoning is consistent with unsafe storage of poisons in places at households where infants and very young children can become accidentally poisoned while exploring their environments. The rural predominance in infants is most likely due to the almost universal exposure of infants to agricultural chemicals often stored in the home.

The male predominance in animal bite is most likely due to the differing gender roles, with females working mainly inside the house. This rural predominance was probably a combination of higher exposure rates to snakes and rabid dogs in rural areas, as well as less availability of antivenom and rabies vaccine in rural areas. A multicentre rabid bite survey in India also established the rural predominance [28].

Our study also revealed electrocution as one of the major causes of childhood death. Childhood deaths due to electrocution are rare and are more likely to occur when children are playing around electrical wires or equipment, and often result from either faulty apparatus, or a lack of understanding of the potential dangers involved [29]. Thus environmental modification along with proper supervision of the younger children is recommended for the prevention of electrocution.

In order to interpret the results of this study, we must explain its limitation as well as strength. This is the largest community-based injury survey in a developing country. Study findings represent the national statistics. In developing countries a large number of injury victims do not attend hospital for treatment, as well as hospital record keeping system is not good enough to document the information. In many developing countries like Bangladesh list of all individual population members (sample frame) is rarely available. Thus, if there is no specific population data a household survey provides population-based injury incidence data and desired information on circumstances in a representative sample [30]. This study captured higher number as study is conducted at household level.

However, the information was based on self-reported data elicited through interviews, which is subject to recall bias [31, 32]. Recall periods of between 1 and 3 months are recommended for survey settings [33]. Thus due to recall period, some moderate injury many not be captured in the study. Moreover the dependency on the description of the interviewers may cause some errors in the determination of mechanism and other risk factors of injury.

Despite its limitations, this study had generated information that could be useful for targeting unintentional child injury prevention at local level. This study described the patterns of unintentional child injuries along with other determinants. It had attempted to identify specific groups of individuals as having a greater risk of experiencing certain types of injuries. This information is important for raising the level of awareness among policy makers and the public in general since the problem of injury receives little attention in most of the developing world including Bangladesh.

## 5. Conclusion

This nationwide study of 171 366 households with 351 651 children under 18 years of age demonstrated that injuries are the major causes of child mortality and morbidity with an overall incidence rate of 1585.9 per 100 000 children per year. Boys had a higher injury incidence rate than that of girls, while rural children had the highest incidence rate. Drowning was the leading cause of injury mortality in children over 1 year of age, and falls were the commonest type of injury morbidity in all age groups. Home and its premises were the most common place for injury while most of the children get injured at the time indoor playing. Hence, there is a need for educational and intervention programs to increase the awareness and understanding of child safety and injury prevention in Bangladesh and to create a safe environment for children. It is also important to involve local experts from the health, education, engineering, legal, and business sectors to determine appropriate steps, intervention techniques, and legislative activities needed to decrease the risk of death or injury from the identified causes.

## Figures and Tables

**Table 1:** Top ten leading causes of morbidity among children (<18 years).

Rank	<1 Year	1–4 Years	5–9 Years	10–14 years	15–17 Years	1–17 Years
1	ARI/Pneumonia (848)	ARI/Pneumonia (1686)	Fall (582)	Fever (505)	Fever (238)	ARI/Pneumonia (2668)
2	Diarrhoeal Diseases (381)	Diarrhoeal Diseases (1287)	Fever (555)	Fall (447)	Fall (169)	Diarrhoeal Diseases (1937)
3	Malnutrition (97)	Burn (580)	ARI/Pneumonia (508)	ARI/Pneumonia (334)	ARI/Pneumonia (140)	Fever (1857)
4	Fever (94)	Fever (559)	Diarrhoeal Diseases (352)	Cut injury (216)	Road Traffic injury (128)	Fall (1600)
5	Fall (53)	Fall (402)	Burn (267)	Diarrhoeal Diseases (210)	Cut injury (94)	Burn (979)
6	Skin Disease (49)	Malnutrition (301)	Measles (264)	Measles (200)	Diarrhoeal Diseases (88)	Measles (828)
7	Measles (37)	Near drowning (297)	Cut injury (261)	Road Traffic injury (188)	Measles (80)	Cut injury (714)
8	ARI and Diarrhoea (36)	Measles (284)	Road Traffic injury (228)	Skin Disease (151)	Jaundice (75)	Road Traffic injury (651)
9	Burn (35)	Skin Disease (216)	Asthma (132)	Animal bite (125)	Malaria (57)	Malnutrition (531)
10	Meningitis (25)	ARI and Diarrhoea (157)	Malnutrition (125)	Jaundice (117)	Burn (42)	Skin Disease (500)

**Table 2:** Incidence of unintentional injuries among children (<18 years) by sex and geographical distribution.

Mechanism of injury	Female			Male			Urban			Rural		
	(*n* = 1878)			(*n* = 3699)			(*n* = 1316)			(*n* = 4261)		
	Rate	95% CI		Rate	95% CI		Rate	95% CI		Rate	95% CI	
		Lower	Upper		Lower	Upper		Lower	Upper		Lower	Upper
RTA	98.1	81.2	118.3	283.3	254.2	315.6	116.1	96.7	139.2	251.6	225.6	280.6
Fall	298.2	268	331.8	642.8	598.5	690.3	320	287	356.7	594.7	554.1	638.3
Falling Object	28.8	20.2	40.9	72.9	58.7	90.4	32.7	23	46.3	65.9	53	81.8
Cut Injury	129.8	110.2	152.7	290.5	261.1	323.3	132.7	111.9	157.3	273.9	246.6	304.1
Burn	252.1	224.4	283.1	323.1	292	357.4	102.6	84.5	124.5	453.3	400.7	472.9
Drowning	129.8	110.2	152.7	151.4	130.5	175.7	29.5	20.3	42.5	229.4	204.5	257.3
Machine	9.8	5.2	18	47.7	36.4	62.3	13.5	7.7	23.2	41.4	31.4	54.4
Electrocution	56.5	44	72.5	100.4	83.6	120.5	25	16.7	37.2	122.1	104.2	143
Animal Bite	62.3	49.1	78.9	141.3	121.1	164.8	62.2	48.4	79.9	134.9	116	156.7
Others	17.9	11.3	28	21.3	14.1	31.9	10.3	5.3	19.2	26.6	18.8	37.5

**Table 3:** Major activities of the children (<18 years) prior to the injury incidence by sex.

Major activities prior to injury	Male	Female	RR	CI at 95%
Work	433.6 (773)	227.3 (394)	1.90	1.69–2.15
Education	11.2 (20)	5.8 (10)	1.94	0.91–4.15
Sport (outdoor)	428.0 (763)	210.0 (364)	2.03	1.80–2.30
Leisure/play (indoor)	467.2 (833)	272.8 (473)	1.71	1.53–1.91
Traveling	315.2 (562)	120.0 (208)	2.62	2.24–3.07
Other	389.3 (694)	235.3 (408)	1.65	1.46–1.87
Unknown	30.3 (54)	12.1 (21)	2.50	1.51–4.14

Total	2074.8 (3699)	1083.3 (1878)	1.90	1.79–2.00
